# Volatile Compounds and Antioxidant Capacity of the Bio-Oil Obtained by Pyrolysis of Japanese Red Pine (*Pinus Densiflora* Siebold and Zucc.)

**DOI:** 10.3390/molecules20033986

**Published:** 2015-03-02

**Authors:** Jayanta Kumar Patra, Sung Hong Kim, Hyewon Hwang, Joon Weon Choi, Kwang-Hyun Baek

**Affiliations:** 1 School of Biotechnology, Yeungnam University, Gyeongsan, Gyeongbuk 712-749, Korea; E-Mail: jkpatra@ynu.ac.kr; 2 Analysis Research Division, Daegu Center, Korea Basic Science Institute, Daegu 702-701, Korea; E-Mail: sunghkim@kbsi.re.kr; 3 Department of Forest Sciences, College of Agriculture and Life Science, Seoul National University, Seoul 151-921, Korea; E-Mail: ch11309@naver.com; 4 Institute of Green-Bio Science and Technology, Seoul National University, Pyeongchang, Gangwon-do 232-916, Korea

**Keywords:** antioxidants, bio-oil, Japanese red pine, pyrolysis, reactive oxygen species

## Abstract

In the present study, sawdust bio-oil (SBO) manufactured by fast pyrolysis of Japanese red pine (*Pinus densiflora* Siebold and Zucc.) sawdust was analyzed for its volatile chemical compound composition and evaluated for its free radical scavenging potential, inhibition of lipid peroxidation and reducing power. Gas chromatography and mass spectroscopy revealed 29 volatile compounds, comprising 97.6% of the total volatile compounds in SBO. The antioxidant potential of SBO in terms of IC_50_ values was 48.44 µg/mL for hydroxyl radical scavenging, 89.52 µg/mL for 1,1-diphenyl-2-picrylhydraxyl radical scavenging, 94.23 µg/mL for 2,2'-azino-bis[3-ethylbenzothiazoline-6-sulphonic acid] radical scavenging, and 136.06 µg/mL for superoxide radical scavenging activity. The total phenol content in SBO was 5.7% gallic acid equivalent. Based on the composition of its volatile compounds, high free radical scavenging potential and antioxidant properties, SBO could be used as a source of antioxidant compounds, flavoring agents and nutraceuticals in the food, pharmaceutical, and cosmetic industries.

## 1. Introduction

Reactive oxygen species (ROS) play important roles in modulation of various physiological and metabolic functions of the body [1]. ROS include free radicals such as hydroxyl (OH^·^) and superoxide anion (O_2_^−^), as well as non-free radicals such as H_2_O_2_ and singlet oxygen (^1^O_2_) [2,3]. To protect against the adverse effects of ROS in living systems, organisms adopt antioxidant defense mechanisms that directly scavenge or prevent the generation of ROS [1]. Nevertheless, excessive generation of ROS in living organisms can overcome these antioxidant defense mechanisms, leading to oxidative stress [4]. This stress then causes damage to different biomolecules such as lipids, proteins, carbohydrates and nucleic acids, leading to various types of degenerative diseases such as cancer, aging, atherosclerosis, cardiovascular disorders, neurodegenerative disorders and inflammation [5,6,7].

ROS can cause lipid peroxidation in foods, which leads to their deterioration and reduced quality [8,9]. To protect food items from the adverse effects of oxidative damage caused by ROS, various synthetic antioxidants such as butylated hydroxytoluene (BHT), butylated hydroxyanisole (BHA) and propyl gallate are commonly added [6]. However, owing to the toxicity and potential health hazards associated with synthetic antioxidants, there has been growing interest in finding natural substances with antioxidant activities [9,10,11].

Plants and their products have been shown to exert antioxidant activity against ROS that allows their effective use as food preservatives, dietary supplements, and food flavoring agents [12,13]. Japanese red pine (*Pinus densiflora* Siebold and Zucc.) is an evergreen tree belonging to the family *Pinaceae* that is widely distributed in Korea [14,15]. The leaves and bark have been used as a source of food additives, food ingredients and folk medicines used throughout Eastern Asia for the treatment of hepatosis, arteriosclerosis and various neurological disorders [14,15]. The leaves of Japanese red pine contain high amounts of volatile compounds with potent biological activities, including antioxidant activities [16]. The bark extracts of *P. densiflora* displayed potential antioxidative and DNA protection activity thanks to their rich content of phenolic compounds, such as procyanidins, phenolic acids and bioflavonoids [17,18]. Hot water extracts of *Pinus* species rich in low molecular weight tannins and phenylpropenoid monomers possess strong antioxidant scavenging potential [19]. The needles of *P. densiflora* contain high amounts of proanthocyanidins and catechins, which have strong antioxidant potential [20]. Bioflavonoids such as phenols and polyphenols (e.g., catechin, taxifolin, epicatechin), condensed flavonoids (e.g., procyanidin, B1, B3 and B7) and phenolic acids (e.g., ferrulic acid, caffeic acid and *p*-hydroxybenzoic acids) extracted from different species of *Pinus* have been reported to possess strong antioxidant potentials [21,22,23].

Sawdust obtained as a byproduct of cutting, drilling, and grinding of wood can be further utilized for the production of naturally occurring safe and potentially useful products that can be utilized in various sectors like foods, cosmetics and pharmaceuticals [24]. Bio-oil (BO) is a liquid product produced by the pyrolysis of biomass materials such as agricultural crops, algal biomass, municipal wastes, and agricultural and forestry by-products (e.g., wood, sugar cane bagasse, rice husks and straw, and coconut fiber) via thermo-chemical/pyrolysis processes [25,26]. BO has been developed for use as an alternative energy source, but also shown to be useful as a source of antioxidant compounds, food additives, and flavor agents [27,28,29,30,31,32,33]. Moreover, BOs manufactured from different sources have been reported to have bactericidal, insecticidal and fungicidal properties [31,34]. BOs are rich in phenolic and tannins, along with other volatile compounds such as furan and furan derivatives, therefore, they have high medicinal potential too [35].

In the present study, the sawdust of Japanese red pine was used to produce a **s**awdust-derived bio-oil (SBO). The chemical composition of the volatile compounds, free radical scavenging and antioxidant potentials of the SBO were then evaluated. Owing to the complicated nature of different types of antioxidants and their reactivity, and since a single antioxidant assay can predict only a reduced view of the antioxidant properties in a sample [36,37], different *in vitro* methods were applied in the present study to evaluate the antioxidant potential of SBO.

## 2. Results and Discussion

### 2.1. Physical Properties of SBO

The elemental composition, water content, viscosity, total acid number (TAN), and higher heating values (HHV) of SBO are given in Table 1. Compared to typical data describing BOs from the other sources [32], the results indicated that the SBO used in this study contained slightly more carbon (50.6%) and less oxygen (41.8%). Accordingly, HHV based on analytical data also slightly increased. The water content and TAN of SBO were similar to those of the other BOs [38], while the viscosity was low (12 cSt), indicating that SBO was easy to handle.

**Table 1:** Physical properties of Japanese red pine sawdust bio-oil (SBO).

Elemental Analysis	Amount
C (%)	50.6
H (%)	6.9
N (%)	0.7
O (by difference) (%)	41.8
Water content (wt%)	23.6
Viscosity (cSt)	12
TAN (mg/g KOH)	74.4
HHV (MJ/kg)	20.7

### 2.2. Chemical Composition of SBO

**Figure 1:**
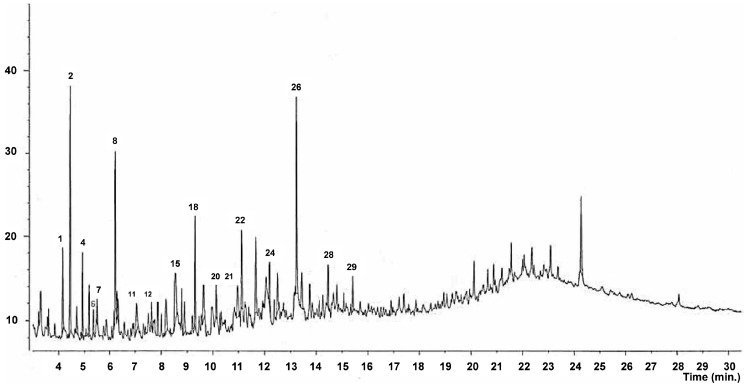
GC-MS spectra of Japanese red pine sawdust bio-oil (SBO).

**Figure 2:**
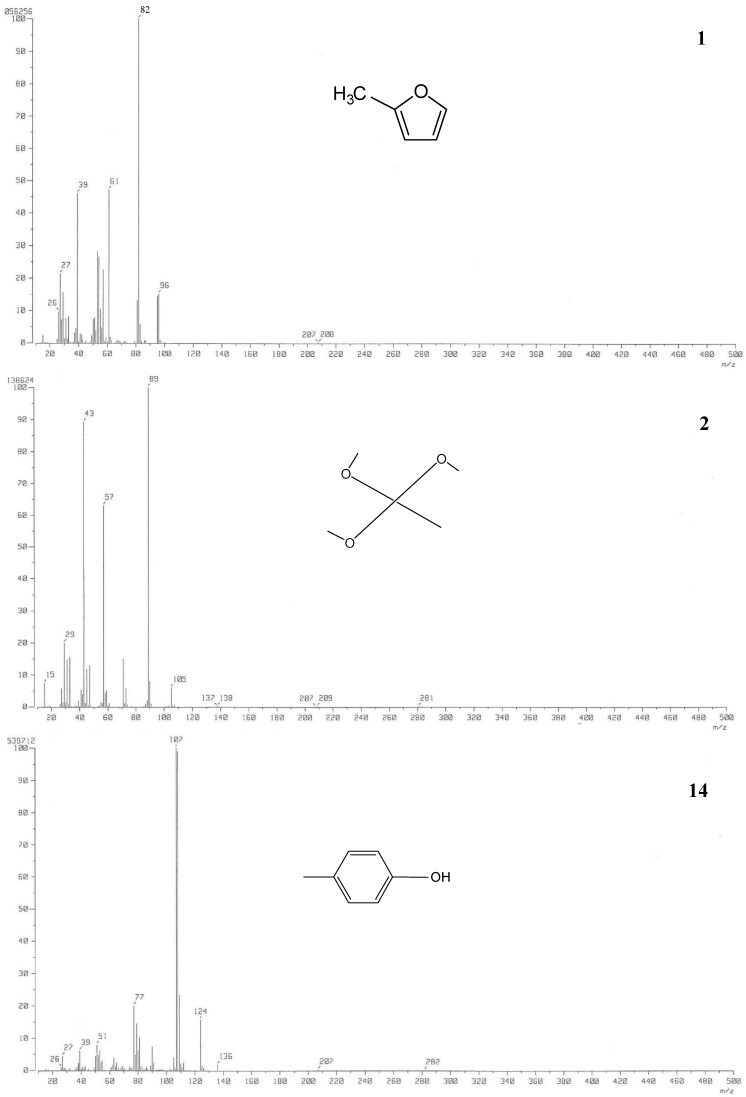

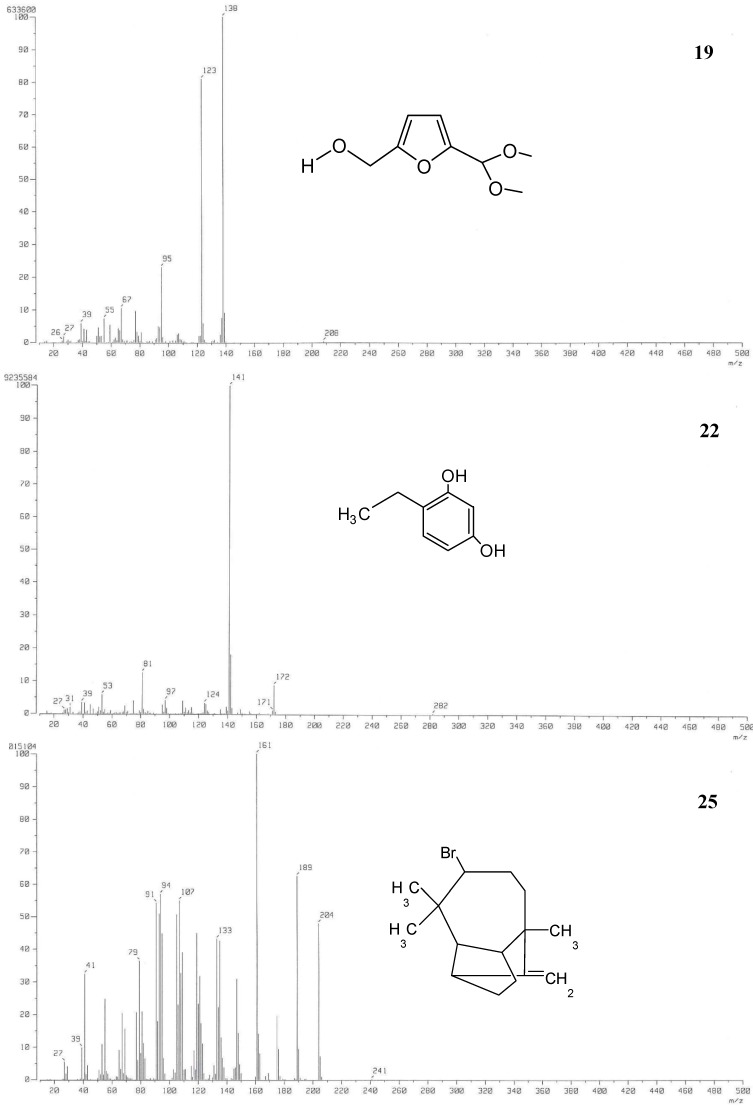
Chemical structure and spectra of some medicinally important compounds present in Japanese red pine sawdust bio-oil (SBO).

GC-MS analysis revealed the chemical composition of volatile compounds in SBO. The chemical profiles of different types of BO based on the GC-MS spectra have previously been reported [39,40,41]. The chemical composition of BO is greatly influenced by the type of raw material used for extraction of BO [42]. For this study, a total of 29 different compounds comprising 97.6% of the volatile compounds in the SBO were selected and identified (Figure 1 and Table 2). The components of SBO mainly comprised aldehydes, alcohols, acids, ether, phenols, and phenol derivatives. Among these, trimethyl orthoacetate (12.8%), 4-oxo-5-methoxy-2-penten-5-olide (8.7%), 1,4-methanoazulene (8.4%), *p*-cresol (6.0%) and benzenemethanamine (5.5%) were the top-five most abundant compounds. Acids, aldehydes, alkanes, benzenes, furans, naphthalenes, alcohols and phenols have also been reported to be present in the pine needles of Japanese red pine [14,15]. Some volatile compounds present in the pine needle powder of Japanese red pine, such as 2-methylfuran, *p-*cresol, phenol, 2(5*H*)-furanone, benzaldehyde and benzenemethanol, were also present in SBO. However, SBO contained additional volatile compounds not present in the pine needle powder (Table 2), possibly due to differences in the type of raw materials used to manufacture the SBO and conversion or degradation of organic matter into numerous small molecules during the fast pyrolysis process used for the manufacture of BO [37]. Volatile compounds such as furan and furan derivatives, ethylresorcinol, cresol, methanoazulene and trimethyl orthoacetate present in SBO have medicinal importance (Figure 2). Furan and furan derivatives form the basic skeleton of numerous compounds with cardiovascular activities. These compounds are widely used for their antiviral, anti-inflammatory, antibacterial, antifungal, antitumor, and antihyperglycemic properties, as well as in the treatment of ventricular and atrial fibrillation [43,44,45]. Ethylresorcinol possesses hypopigmentary and antioxidant properties [46], and cresols are used in the preparation of antioxidants and as abortives, diaphoretics, and emmenagogeous drugs [43,44,47,48]. 4-oxo-5-methoxy-2-penten-5-olide previously identified as a component of honey has potential antiproliferative potential [48]. Further, compounds present in SBO in higher concentrations can be upgraded to other useful compounds by the process of molecular distillation technology and can be utilized in pharmaceutical industries [32]. *p-*Cresol, found in high concentration (6.0%), can be converted to other usable antioxidants and diphenol antioxidants with relatively low toxicity [27] by the *tert*-butylation procedure using a Bronsted acid functionalized ionic liquid, and those conversion products can be potentially used in the food, pharmaceutical and cosmetic industries [30]. Trimethyl orthoacetate present in SBO can be used in the pharmaceutical industry for the esterification of ferulic acid to produce more potent antioxidants [28,29].

**Table 2:** Major components of Japanese red pine sawdust bio-oil (SBO) based on GC-MS analysis.

No.	SI ^a^	RT ^b^	Compound ^c^	Composition (%)	Identification Method ^d^
1	554	4.15	2-Methylfuran	4.28	EI-MS
2	676	4.45	Trimethyl orthoacetate	12.78	EI-MS
3	773	4.70	Acetol acetate	1.30	EI-MS
4	812	4.92	2,5-Dimethoxytetrahydrofuran	2.92	EI-MS
5	823	5.35	2-Methyl-2-cyclopentenone	1.17	EI-MS
6	897	5.48	2(5*H*)-Furanone	1.01	EI-MS
7	782	6.23	4-Oxo-5-methoxy-2-penten-5-olide	8.72	EI-MS
8	533	6.30	Erythrite tetramethyl ether	1.38	EI-MS
9	534	6.33	2,3,4-Trimethylfuran	1.07	EI-MS
10	803	7.05	Phenol	2.19	EI-MS
11	600	7.63	Hexanal dimethyl acetal	0.95	EI-MS
12	563	7.88	1,1,N,N-(tetramethylbuta)-1,3-diene-4-amine	2.08	EI-MS
13	891	8.18	*o*-Cresol	2.79	EI-MS
14	763	8.54	*p*-Cresol	6.02	EI-MS
15	645	8.79	Benzaldehyde dimethyl acetal	1.18	EI-MS
16	455	8.89	Butanoic acid	1.68	EI-MS
17	577	9.31	Hexanalldimethyl acetal	4.90	EI-MS
18	847	9.63	2,5-Xylene	3.20	EI-MS
19	716	10.13	4-Ethylresorcinol	1.32	EI-MS
20	809	10.96	*p*-Ethylanisole	2.62	EI-MS
21	902	11.11	5,4-Dimethyl-2-methylbibenzyl	4.10	EI-MS
22	684	11.66	5-(Hydroxymethyl)-2-(dimethoxymethyl) furan	4.75	EI-MS
23	671	12.19	1,3-Bis(trimethylsiloxy)benzene	2.36	EI-MS
24	613	12.49	2-Methoxy-6-(1-propenol) phenol	2.48	EI-MS
25	871	13.24	1,4-Methanoazulene	8.44	EI-MS
26	357	13.44	6-Methyl-4-indanol	2.60	EI-MS
27	626	14.46	4-(Phenylmethyl)benzenemethanol	2.35	EI-MS
28	748	15.42	Napthalene	1.50	EI-MS
29	493	24.28	Benzenemethanamine	5.48	EI-MS

^a^ SI-Library search purity value; ^b^ RT-Retention time; ^c^ Compounds listed in order of elution; ^d^ Identification based on computer matching of electron ionization mass spectra using Wiley and NIST libraries for the GC-MS system.

SBO can be utilized as food additive giving smoked, roasted and grilled flavors to food products [49]. SBO was also found to be rich in various types of volatile compounds with medicinal potential, therefore, further investigation of its role as a source of antioxidant compounds could be beneficial for its application in the food sector as a food additive, flavoring agent, food preservation and possibly in pharmaceutical and cosmetic industries.

### 2.3. DPPH Free Radical Scavenging Activity of SBO

The DPPH free radical scavenging potential of SBO is presented in Table 3. The concentration of SBO and gallic acid exerting 50% DPPH scavenging potential is known as its IC_50_ value and was found out to be 89.52 µg/mL and 21.73 µg/mL respectively (Table 3). There was a significant decrease in the concentration of DPPH radical as the concentration of SBO increased due to its ability to scavenge free radicals. DPPH radical is considered a model liphophilic radical; therefore, the scavenging activity of SBO is attributed to its ability to donate hydrogen ions or electrons to DPPH to neutralize free radicals [1]. However, its effects may be due to its inhibitory effect on the free radicals, particularly the peroxy radicals, which are the propagators of lipid peroxidation [50].

**Table 3:** Free radical scavenging potential of Japanese red pine sawdust bio-oil (SBO).

Sample	DPPH Free Radical Scavenging	ABTS Free Radical Scavenging	Hydroxyl Radical Scavenging	Superoxide Anion Scavenging	Nitric Oxide Scavenging
SBO	89.52 *	94.23	48.44	136.06	362.45
Gallic acid	21.73	4.48	3.99	25.08	52.59

* Data are expressed in terms of IC_50_ values (µg/mL).

### 2.4. ABTS Free Radical Scavenging Activity of SBO

The generation of ABTS radical cations is the basis of an important spectrophotometric method for determination of the antioxidant potential of various natural substances [51]. The ABTS free radical scavenging potential of SBO is presented in Table 3. The IC_50_ value of SBO and the reference compound, gallic acid was found out to be 94.23 µg/mL and 4.48 µg/mL respectively (Table 3). Various factors, including solubility of SBO, bioactive compounds present in it and the stereoselectivity of the radicals might be responsible for its ABTS radical scavenging potential [52,53]. Though SBO exerted less scavenging activity than the reference compound, it might still be useful as a valuable antioxidant resource with various applications, especially in the food and cosmetic industries considering its cost effectiveness.

### 2.5. Hydroxyl Radical Scavenging Activity of SBO

The hydroxyl radical scavenging activity of SBO in terms of IC_50_ was 48.44 µg/mL, while that of gallic acid was 3.99 µg/mL (Table 3). Hydroxyl radical is a highly reactive oxygen free radical that is responsible for various types of cellular damage and lipid peroxidation in living organisms and foods [54]. Accordingly, SBO with high hydroxyl radical scavenging potential can further be exploited for its potential application as a food preservative to protect food products from oxidative damage-related issues. Nowadays, consumers are more concerned about the use of synthetic chemicals by food industry in flavoring, preserving and processing [55,56], therefore, the strong hydroxyl potential of SBO could make it an alternative for use as an antioxidant by the food industry.

### 2.6. Superoxide Anion Scavenging Activity of SBO

As shown in Table 3, SBO and gallic acid scavenged superoxide anions effectively. The IC_50_ value of SBO and gallic acid was 136.06 µg/mL and 25.08 µg/mL, respectively. Superoxide anions are the precursors to reactive free radicals, which deteriorate various biological macromolecules, thereby inducing tissue damage [57]. Superoxide anion has been implicated in several pathophysiological processes owing to its transformation into more reactive species such as hydroxyl radicals, which are responsible for lipid peroxidation, and singlet oxygens, which cause oxidative damage to DNA, lipids and proteins [50,58]. The effective superoxide scavenging potential of SBO could make it a potential ingredient for the food, pharmaceutical and cosmetic industries as an effective antioxidant compound.

### 2.7. Nitric Oxide Scavenging Activity of SBO

SBO and gallic acid showed IC_50_ values of 362.45 µg/mL and 52.59 µg/mL, respectively (Table 3). SBO showed comparatively less nitric oxide scavenging potentials than gallic acid. Under aerobic conditions, endogenously generated nitric oxide is a very unstable species involved in the regulation of many physiological processes that lead to inflammation, vasodilatation and immune regulation; thus, it is associated with cancer and inflammatory conditions [59]. The activity of SBO in the present study may have been due to the presence of antioxidant compounds that compete with oxygen to react with nitric oxide and inhibit the generation of nitrite. Nitric oxide produced by UV radiation mediates a remarkably diverse and complex range of biological functions in the skin. Especially, with the presence of superoxide radical nitric oxide can form highly reactive peroxynitrite, leading to nitrosation of tyrosine, breakage of DNA strand and skin aging [60,61]. Thus the potential nitric oxide scavenging effect of SBO could make it a suitable candidate for cosmetic industry in the production of sun cream lotions and anti-aging formulations [62].

### 2.8. Inhibition of Lipid Peroxidation Activity of SBO

**Figure 3:**
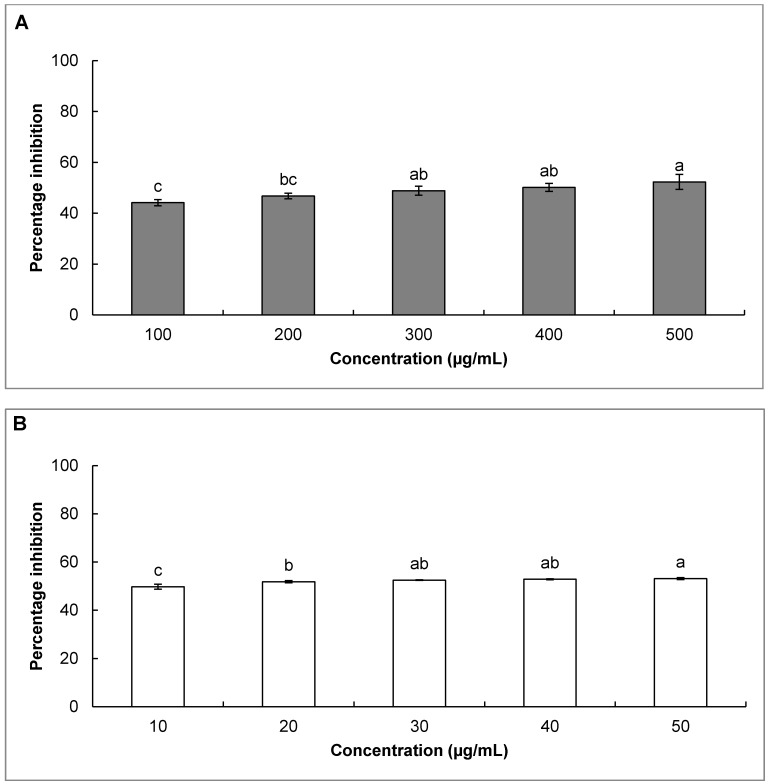
Inhibition of lipid peroxidation by (**A**) Japanese red pine sawdust bio-oil (SBO) and (**B**) gallic acid as a reference. Columns with different superscript letters indicate a significant difference at *p* < 0.05.

The inhibition of the lipid peroxidation potential of both SBO and gallic acid is presented in Figure 3. Both SBO and gallic acid showed significant inhibition of lipid peroxidation of 44.1% and 49.8% at 100 µg/mL and 10 µg/mL, respectively. There is evidence that lipid peroxidation is responsible for different types of food deterioration processes affecting color, texture, flavor, nutraceutical and nutritional values of food and various food products [24]. The modifications of low density lipoproteins by oxidative process play a major role in causing atherosclerosis [24,63], but this modifications of low density lipoproteins can be overcome by the addition of antioxidants [64].

Due to the high capacity for the inhibition of lipid peroxidation by SBO, it can be used as an alternative to synthetic preservatives and antioxidants commonly used by food industries to preserve foods and increase their shelf life and may also have applications in the development of drugs related to oxidative stress and lipid peroxidation. The skin is rich in lipids which are sensitive to oxidative processes [65], thus SBO with higher lipid peroxidation inhibitory activity could be utilized for the formulation of various skin care, anti-aging and anti-wrinkle products in the cosmetic industry.

### 2.9. Reducing Power and Total Phenol Content of SBO

**Figure 4:**
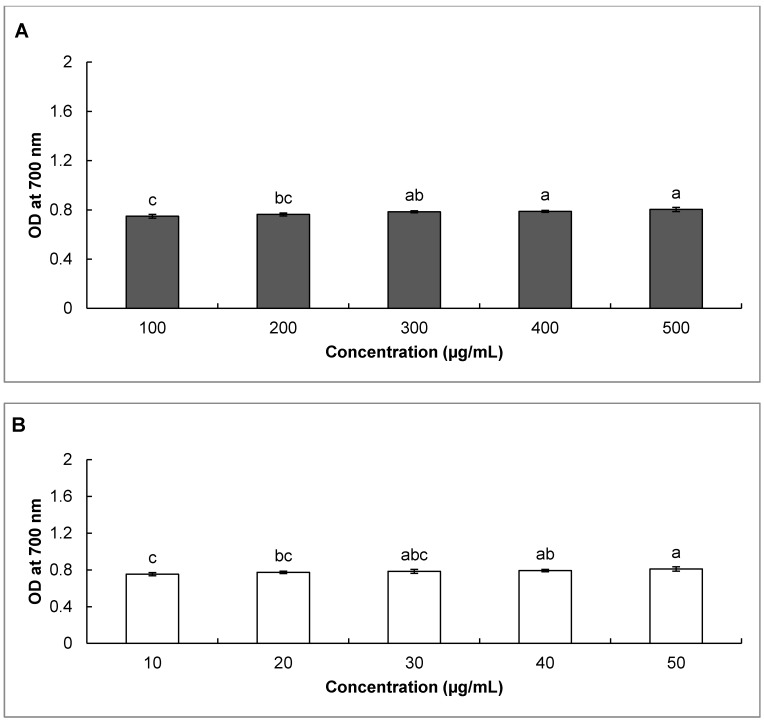
Reducing power of (**A**) Japanese red pine sawdust bio-oil (SBO) and (**B**) gallic acid as the reference. Columns with different superscript letters indicate a significant difference at *p* < 0.05.

Another reaction pathway in the donation of electrons is the reduction of an oxidized antioxidant molecule to regenerate the active reduced antioxidants [36]. Determination of reducing power is an important aspect for estimation of antioxidant potential of any sample. As shown in Figure 4, SBO and gallic acid displayed high reducing power as evident from the increased absorbance values, indicating its strong reduction capability. The antioxidant potential of any compound is mainly due to its redox properties, which play an important role in neutralizing free radicals and quenching singlet oxygen [66]. It has been suggested that there is a direct relationship between antioxidant potential and the reducing power of certain biological samples [67].

The total phenolic content of SBO was equivalent to 5.7% gallic acid based on the calculation curve (data not shown). On an average, the phenol content of pyrolysis liquid is only 2%–5% [68], however, SBO contained 5.7% of phenol content, which content can be considered as to be rich in phenolic content. The antioxidant potentials of different plant species were due to the presence of phenolic compounds [69].

The phenolic compounds in food have also been demonstrated to possess potential antioxidant activity [70]. Thus the antioxidant potential of SBO in the present study may be attributed to the presence of high levels of phenolic compounds that display potent antioxidant properties. The phenolic compounds with reducing potential and antioxidant property can inhibit the formation of superoxide anion radicals [71], thus the high amount of total phenol content of SBO could be correlated to its antioxidant potential. Food, pharmaceutical and cosmetic industries are increasing their interest in polyphenolic compounds, which have been shown to neutralize the effect of adverse free radicals with potential antioxidant properties and to prevent many diseases related to oxidative stress, such as aging, cancer, degenerative diseases, *etc.* [62,72].

Extracts of *P. densiflora* have been reported to possess strong antioxidant potential due to the presence of phenolic compounds such as catechin, taxifolin, epicatechin, condensed flavonoids (e.g., procyanidins), phenolic acids (ferrulic acid, caffeic acid, *p*-hydroxybenzoic acids), and tannins [18,19,20,21,22,23]. Thus the antioxidant potential of SBO could be attributed to the rich presence of phenolic compounds originated from the sawdust of *P. densiflora* and other chemical compounds produced from the manufacturing process. SBO with rich phenolic compounds could be considered as a potential candidate for the formulation of new nutraceuticals and skin care products for the cosmetic industries.

Certain antioxidant compounds, including phenolic antioxidants and tocopherols, have been shown to undergo loss of activity at high concentrations and become prooxidative or reactive [73]. The direct addition of antioxidant compounds to food products in higher doses results in a rapid depletion of the antioxidants in the long run. SBO could be an economic solution as an additive for developing polymer packaging materials which can deliver antioxidant compounds in a controlled manner throughout the product’s shelf life.

## 3. Experimental Section

### 3.1. Materials

#### 3.1.1. Chemicals and Instruments

All chemicals such as 2,2'-azino-bis[3-ethylbenzothiazoline-6-sulphonic acid] (ABTS), diphenyl-2-picrylhydraxyl (DPPH), sodium nitroprusside (SNP), Griess reagent, nitroblue tetrazolium (NBT), phenazine methosulphate (PMS), trichloroacetic acid (TCA), ferric chloride, potassium ferricyanide and gallic acid were of analytical grade and purchased from Sigma Aldrich (St. Louis, MO, USA). Spectrophotometric measurements were performed using a 96-well multimode microplate reader (Infinite M200 PRO NanoQuant, Tecan, Mannedorf, Switzerland).

#### 3.1.2. Production of BO from Japanese Red Pine Sawdust

Japanese red pine was obtained from Korea Forest Research Institute in the form of bark free wood chips, which were ground to sawdust and sieved to a particle size of 0.5 mm. After being dried to *ca*. 7% moisture content, the sawdust was used as raw material for the manufacture of SBO in a lab-scale fluid-bed fast pyrolysis procedure. Fast pyrolysis of the sawdust was then performed at 500 °C in a nitrogen atmosphere with a pyrolysis product residence time of 1.3 s [74]. The manufactured SBO was kept at 4 °C until further analysis.

### 3.2. Methods

#### 3.2.1. Physical Analysis of SBO

SBO obtained from the fast pyrolysis process was characterized by several analytical methods. The water content was determined using a Karl-Fisher titration unit with hydranal composite 5 solutions. The composition of carbon, hydrogen, and nitrogen present in the manufactured SBO was analyzed by an elemental analyzer (FLASH 2000 CHNS/O Analyzers, Thermo Fisher Scientific, Waltham, MA, USA) and the ratio of oxygen was calculated based on the difference. The higher heating values (HHV) of SBO were estimated according to the Sheng and Azevedo’s correlation [75]. Total acid number (TAN) and kinetic viscosity at 40 °C were determined by a titrator (848 Titrino plus, Metrohm, Herisau, Switzerland) using 0.1 M KOH and a capillary viscometer, respectively.

#### 3.2.2. Chemical Analysis of the Volatile Compounds in SBO

The chemical composition of volatile compounds in SBO was determined by gas chromatography and mass spectra analysis using a GC-MS system (Jeol JMS 700 MStation, Jeol USA, Peabody, MA, USA) equipped with an Agilent 6890N GC DB-5 MS fused silica capillary column (30 m × 0.25 mm i.d. and film thickness of 0.25 µm). For GC-MS detection, an electron ionization system with ionization energy of 70 eV was used and helium was applied at a constant flow rate of 1 mL/min as the carrier gas. The temperature of the injector and MS transfer line was set at 280 °C and 250 °C, respectively. Initially, the oven temperature was maintained at 50 °C for 2 min, after which it was increased to 250 °C at a rate of 10 °C/min, where it was held for 10 min. Samples (1 µL of 100 times-diluted samples in methanol) were injected manually in splitless mode through the injector. The relative percentages of the constituents of SBO were expressed as percentages calculated by normalization of the peak area. Identification of various volatile compounds in SBO was based on the GC retention time on a DB-5 capillary column relative to computer matching of mass spectra using the Wiley and National Institute of Standards and Technology libraries for the GC-MS system [76]. The chemical structures of some of the medicinally important compounds were drawn using the ACD Chemsketch software [77].

#### 3.2.3. Radical Scavenging Potential of SBO

The radical scavenging and antioxidant potential of the crude SBO was determined by DPPH radical, ABTS radical, hydroxyl radical, superoxide anion and nitric oxide scavenging assays, as well as lipid peroxidation inhibition and reducing power assays.

##### DPPH Radical Scavenging Activity of SBO

The DPPH free radical scavenging potential of SBO was determined as per the standard procedure, with slight modification [78]. Briefly, 50 µL of different concentrations of SBO (100–500 µg/mL) or gallic acid (10–50 µg/mL) were added to 50 µL of 0.1 mM DPPH in methanol in a 96-well flat bottom microplate (SPL Life Sciences, Pocheon-si, Gyeonggi-do, South Korea). A mixture of 50 µL each of methanol and 0.1 mM DPPH solution was taken as the control. The reaction mixture was incubated at 37 °C for 30 min in darkness with shaking at 150 rpm, after which the absorbance of the sample was taken at 517 nm using the 96-well microplate reader. The concentration of SBO to scavenge 50% of DPPH radical was calculated as IC_50_ values from the regression analysis.

##### ABTS Radical Scavenging Activity of SBO

The ABTS free radical scavenging potential of the SBO was determined by the standard procedure, with slight modification [51]. Prior to the experiment, stock solutions of 7.4 mM ABTS solution and 2.6 mM potassium persulfate solution were prepared in separate bottles and kept in darkness. The required amount of the two stock solutions was mixed equally and allowed to react for 12 h in darkness to manufacture the ABTS working solution. Next, 15 µL of different concentrations of SBO (100–500 µg/mL) or gallic acid (10–50 µg/mL) was added to 285 µL of the ABTS working solution and kept in the dark for 2 h. Reaction mixture amended with 15 µL of methanol and 285 µL of the ABTS working solution was taken as the control. The absorbance of the reaction mixture was taken at 730 nm using a 96-well microplate reader. The result was represented in terms of IC_50_ values (concentration of SBO scavenging 50% of ABTS radicals).

##### Hydroxyl Radical Scavenging Activity of SBO

The hydroxyl radical scavenging potential of SBO was determined by a standard procedure [79]. The total volume of the reaction mixture (240 µL) contained 3 mM 2-deoxyribose, 20 mM potassium phosphate buffer (pH 7.4), 0.1 mM ferric chloride, 0.1 mM ethylenediamine tetraacetic acid, 2 mM hydrogen peroxide, 0.1 mM ascorbic acid, and 40 µL of various concentrations of SBO (100–500 µg/mL) or gallic acid (10–50 µg/mL). After incubating the reaction mixture at 37 °C for 45 min, 40 µL each of 2.8% TCA and 0.5% TBA in 0.025 M sodium hydroxide solution containing 0.02% BHA were added and incubated at 90 °C for 15 min to develop a pink color. The mixture was then allowed to cool, after which the absorbance was measured at 532 nm. The reaction mixture amended with 40 µL methanol was taken as a control. The hydroxyl radical scavenging potential of SBO was represented in terms of IC_50_ values, which was calculated by the regression curve analysis.

##### Superoxide Anion Scavenging Activity of SBO

The superoxide anion scavenging potential of SBO was evaluated by measuring the reduction of NBT [80]. The total volume of the reaction mixture (100 µL) contained 40 µL of 0.02 M phosphate buffer (pH 7.4), 10 µL of 73 µM NADH, 10 µL of 50 µM NBT, 10 µL of 15 µM PMS and 30 µL of various concentrations of SBO (100–500 µg/mL) or gallic acid (10–50 µg/mL) as the standard. The mixture was incubated for 60 min at room temperature, after which the quantity of formazan generated was determined by taking the absorbance of the reaction mixture at 560 nm. Reaction mixture amended with only 30 µL of methanol was taken as a control. The superoxide radical scavenging potential of SBO was calculated in terms of IC_50_ value, which was calculated by the regression curve analysis.

##### Nitric Oxide Scavenging Activity of SBO

The nitric oxide radical scavenging potential of SBO was determined by the standard method [24]. The reaction mixture of 200 µL consisted of 100 µL of different concentrations of SBO (100–500 µg/mL) or gallic acid (10–50 µg/mL) and 100 µL of 10 mM sodium nitroprusside in phosphate buffer saline (pH 7.4). The mixture was incubated at 37 °C for 60 min in light, after which 75 µL aliquots of the reaction mixture were mixed with 75 µL of Griess reagent (1.0% sulfanilamide and 0.1% napthyl ethylenediamine dihydrochloride) and then incubated at 25 °C for 30 min in darkness. The absorbance was then measured at 546 nm. Reaction mixture amended with only 100 µL methanol was taken as a control. The nitric oxide scavenging potential of SBO was represented by IC_50_ values, which was calculated by the regression curve analysis.

#### 3.2.4. Inhibition of Lipid Peroxidation Activity of SBO

The inhibition of lipid peroxidation by SBO was assayed by the Fe^3^^+^/ascorbic acid-dependent non enzymatic lipid peroxidation method using bovine brain extract as previously described, with slight modification [81]. Briefly, a 100 µL reaction mixture consisting of 30 µL of SBO (100–500 µg/mL) or gallic acid (10–50 µg/mL) at different concentrations, 50 µL of bovine brain phospholipids (5 mg/mL), 10 µL of 1 mM FeCl_3_ and 10 µL of 1 mM ascorbic acid in 20 mM phosphate buffer. The mixture was incubated at 37 °C for 1 h. After incubation, 100 µL each of 1% TBA and 30% TCA acid and 10 µL of 4% BHT was added and the sample was heated in boiling water for 20 min. The absorbance was then measured at 532 nm using the 96-well microplate reader, after which the percent inhibition of lipid peroxidation was calculated using the following formula:

Inhibition of lipid peroxidation (%) = [(A_0_ − A_t_)/A_0_] × 100

where, A_0_ is the absorbance of the control and A_t_ is the absorbance of the treatment.

#### 3.2.5. Reducing Power of SBO

The Fe^3^^+^ reducing power of SBO was determined as previously described [82]. Briefly, 50 µL of SBO (100–500 µg/mL) or gallic acid (10–50 µg/mL) was mixed with 50 µL of 0.2 M phosphate buffer (pH 6.6) and 50 µL of 1% potassium ferricyanide. The mixture solution was then incubated at 50 °C for 20 min in darkness. After incubation, 50 µL of 10% TCA was added to terminate the reaction and the mixture was centrifuged at 3000 rpm for 10 min. Next, 50 µL of supernatant was mixed with 50 µL of distilled water and 10 µL of 0.1% FeCl_3_ solution and incubated for 10 min at room temperature. Finally, the absorbance was measured at 700 nm and the result was expressed in terms of absorbance at 700 nm.

#### 3.2.6. Total Phenolic Content of SBO

The Folin-Ciocalteu method was used to measure the total phenolic content of SBO [83]. Briefly, 50 µL of SBO corresponding to 0.1 mg/mL was mixed with 50 µL of 50% Folin-Ciocalteu reagent. After incubating the mixture at 25 °C for 5 min in darkness, 100 µL of 20% Na_2_CO_3_ solution was added and the sample was incubated for an additional 20 min at 25 °C for 20 min. Finally, the absorbance of the reaction mixture was measured at 730 nm and the total phenolic content was determined from the standard calibration curve of gallic acid using a concentration range of 5–50 µg/mL.

#### 3.2.7. Statistical Analysis

All results were expressed as the mean ± standard deviation (SD) based on three independent replicates. Statistical interpretation of the results to test the significance of differences between means obtained among the treatments was conducted by one-way analysis of variance (ANOVA) followed by Duncan’s test at the 5% level of significance (*p <* 0.05) using the Statistical Analysis Software (SAS) (Version: SAS 9.2, SAS Institute Inc., Cary, NC, USA).

## 4. Conclusions

The results of the present study demonstrate that SBO manufactured by pyrolysis of Japanese red pine sawdust contains phenolic compounds, acids, furans and other aromatic compounds. It possesses significant antioxidant potential that occurs via free radical scavenging, inhibition of lipid peroxidation and reducing power. SBO contains various useful compounds that could be used as a flavoring agent and food additive giving smoked, roasted and grilled flavor to food products. Apart from this, it can be utilized by the packaging industry as an additive for polymer packaging materials and by the pharmaceutical and cosmetic industries for use in preparation of useful compounds, medicines and sunscreens, and anti-aging, anti-wrinkle lotions with antioxidant potential.

## Authors Contributions

J.K.P. has contributed in performing experiments and writing the manuscript. S.H.K. and H.H. contributed to the chemical and physical analysis of samples. K.H.B. and J.W.C. contributed in planning and designing the study and analysis of data. All authors participated in drafting the manuscript; they have read and approved the final manuscript.
